# Orgasmic Meditation, Sleep, and Next-Day Somatic Distress: A 30-Day Observational Study

**DOI:** 10.7759/cureus.118014

**Published:** 2026-10-16

**Authors:** Caroline Griggs

**Affiliations:** 1 Research, Institute of OM Foundation, Santa Rosa, USA

**Keywords:** meditation, observational study, orgasmic meditation, sexual wellness, sleep quality, well-being, women's health

## Abstract

Background: Orgasmic Meditation (OM) is a structured attention-training practice conducted between two people who follow a predefined set of detailed instructions. One person, the stroker, gently strokes the clitoris of the other person, the strokee, for 15 minutes while both place their attention on the point of contact and notice what they feel. Climax is not the goal. This study examined whether OM practice was associated with app-assessed sleep and next-morning Tumescence Survey responses.

Methods: In this 30-day observational study, 41 experienced OM practitioners enrolled, and 36 contributed analyzable data, yielding 1,026 participant-nights. The Sleep Cycle Premium application assessed sleep quality, duration, regularity, and time to sleep onset. Each morning, participants reported whether they had practiced OM the previous day and completed the 15-item Tumescence Survey. Linear mixed-effects models accounted for repeated nights from the same participants and separated within-person from between-person associations.

Results: OM practice was not significantly associated with sleep quality (b = -1.87 index points; 95% CI: -3.96 to 0.21; p = .079), sleep duration (-10.11 minutes; 95% CI: -22.37 to 2.15; p = .106), time to sleep onset (+2.01 minutes; p = .225), or sleep regularity (+0.05; p = .948). On mornings after OM, participants reported less dissociation from their bodies and less agitation. These two item-level associations remained significant after correction for 15 comparisons. Craving and irritability scores were slightly lower after OM, but the differences were not statistically significant. Factor 2, which combined craving, physical discomfort, agitation, and dissociation, was lower after OM in the primary model (b = -0.77; 95% CI: -1.17 to -0.37; p < .001). The estimate weakened when the OM association was allowed to vary among participants (b = -0.67; 95% CI: -1.36 to 0.02; p = .056). Sleep did not mediate or modify this association. With OM practice and sleep duration in the same model, a day of OM was associated with the same reduction in Factor 2 as 3.8 additional hours of sleep (95% CI: 1.8 to 9.5), and with the same reduction in the total survey score as 1.7 additional hours (95% CI: 0.2 to 3.9). Climax was reported on 19.4% of OM days and was not associated with sleep outcomes within OM days.

Conclusions: OM practice was not associated with app-assessed sleep in this sample. On mornings after OM, participants reported less dissociation from their bodies and less agitation, and these differences were not explained by sleep duration. The difference associated with an OM day was comparable in size to the difference associated with 3.8 additional hours of sleep for Factor 2 and 1.7 additional hours for the total survey score. The estimates weakened when the OM association was allowed to vary among participants. This comparison describes the relative size of observational associations and does not show that OM reduces sleep need. The sample was small and highly selected, and neither the sleep application nor the Tumescence Survey has been validated for this use. Controlled studies using validated measures are needed.

## Introduction

Sleep and sexual function are each substantial contributors to quality of life, and each has well-documented associations with mental and physical health [1-4]. Insufficient sleep affects roughly one in three adults in the United States [5], and poor sleep quality has been associated with increased risk of cardiovascular disease, metabolic disease, obesity, and depression [6]. Sexual activity is widely believed to promote sleep [7]. Direct evidence, however, is sparse.

Costa et al. assessed 275 Portuguese adults (169 women) at a single time point and found that poorer subjective sleep quality was associated with greater unstimulated sexual arousal, an association whose strength depended on salivary testosterone; sleep quality was uncorrelated with sexual desire or sexual frequency over the preceding month [8]. Kalmbach et al., using a 14-day daily diary in 171 women, found that longer sleep on a given night predicted greater next-day sexual desire and a 14% increase in the odds of partnered sexual activity per additional hour of sleep [9]. These findings appear to conflict. The second study also contained both directions: within individuals, longer sleep predicted poorer next-day genital arousal, whereas across individuals, women who habitually slept longer reported better genital arousal.

Later reviews have not resolved whether sexual activity itself improves sleep. A systematic review of 13 studies found some evidence linking sexual function, sexual satisfaction, and masturbation with sleep quality, but no association with sexual frequency; studies using subjective sleep outcomes were more likely to report an association than studies using objective measures [10]. Meditation research has more often asked whether practice improves sleep disturbance than whether it reduces sleep need. Across 18 randomized trials, mindfulness improved sleep quality compared with nonspecific attention controls but not compared with established sleep treatments [11]. Together, this literature supports separating subjective benefit, objective sleep, and daytime function rather than treating them as the same outcome.

When repeated observations from the same individuals are pooled without regard to which person contributed them, the resulting estimate mixes two different questions: whether a person differs from their own usual state on days they engage in a behavior, and whether people who engage more often differ from people who engage less. The pooled estimate mostly reflects the second, between-person comparison, and that comparison is confounded by every stable characteristic that distinguishes the two groups [12,13]. We therefore designed the present analysis to estimate the two levels separately.

The practice examined here is Orgasmic Meditation (OM), a structured attention-training practice conducted between two people who follow a predefined set of detailed instructions. One person, the stroker, gently strokes the clitoris of the other person, the strokee, for 15 minutes while both place their attention on the point of contact and notice what they feel. Climax is not the goal [14]. The sequence of steps is described in the Methods.

Partnered genital stimulation and contemplative practice are each long established. OM combines them in a single manualized, time-limited protocol, and that protocol entered the peer-reviewed literature only recently. Five prior empirical studies have examined OM, concentrated on neuroimaging, subjective experience, and physiological response [14-18], and none has examined its relationship to sleep. Those studies share authors and funding with the present one, so independent replication of any OM finding remains important.

OM is easier to study than most sexual activity. Its duration is fixed, its sequence of steps is manualized, participant roles are defined, and the practice is explicitly goalless with respect to orgasm. In ordinary sexual activity, duration, activity, and intent vary together from one occasion to the next; in OM, the protocol fixes all three. In a neuroimaging study, brain activity after OM overlapped with the activity reported in other meditative practices [14], and in a survey, a large majority of practitioners regarded the practice as more similar to meditation than to sex [15].

The Tumescence Survey is a 15-item instrument developed to capture symptoms and behaviors that practitioners commonly describe as changing with OM practice. Tumescence in this context denotes a state of energetic congestion; higher scores indicate greater endorsement of psychological distress, dissociation, physical discomfort, agitation, and craving. Because the instrument had not been validated before this study, its scores are treated as descriptions of responses to these items rather than as an established measure of well-being.

The primary objective was to examine the association between OM practice and app-assessed sleep on the following night, with sleep quality as the primary outcome. Secondary objectives were to examine the association between OM practice and next-morning Tumescence Survey responses, to describe the instrument's psychometric properties, and to determine whether sleep accounted for any portion of the association between OM practice and those responses. No prior evidence addressed genital stimulation and app-assessed sleep, so hypotheses regarding sleep were non-directional. The practice occurred naturally and was observed rather than assigned, so the results describe associations and cannot establish causal effects.

## Materials and methods

Participants

Participants were a convenience sample, recruited from those available rather than selected at random, of regular OM practitioners. They were identified from the mailing list of OneTaste, a company that trains people in the practice of OM, or through practicing OM while sheltering in place at a retreat center in Northern California. Participants were not paid. Potential participants received an email with a flyer that described the study and gave contact information for the study coordinator. All enrolled participants were ≥ 18 years old, practiced OM at least once per week on average, and were willing to use the Sleep Cycle app for 30 continuous nights during the study period. Participants were required to be conversant in English. There were no exclusions by race, ethnicity, or sexual orientation. The IntegReview Institutional Review Board approved the study. Signed informed consent was obtained from all participants before any study activities. The study was registered with clinicaltrials.gov before recruitment began (NCT04455971). Recruitment and data collection took place between October 6, 2020, and April 15, 2021.

Study design overview

This 30-day observational study compared each participant with themselves across nights (a within-subjects design) to evaluate associations between the frequency and characteristics of OM practice, app-based measures of sleep quality, and a questionnaire-based assessment of next-morning symptoms and experiences that may relate to subjective well-being. Participants tracked sleep every night using the Sleep Cycle app and answered a series of questions each morning about the prior day, including (1) whether the participant had engaged in an OM session the prior day; (2) the number of OM sessions; 3) whether the participant was a stroker or strokee; (4) whether climax had occurred; and 5) whether the participant had engaged in physical exercise. In addition, participants completed the 15-item Tumescence Survey. Sleep parameters were measured every night with the Sleep Cycle Premium app, which ran on a cell phone placed next to the sleeper's bed with the device microphone facing the bed. Study data were collected and managed using REDCap electronic data capture tools hosted at the Institute of OM Foundation [19,20].

The protocol neither specified nor required OM practice; participants practiced when and as often as they chose. Study personnel recorded the amount of OM practice during the study period and did not attempt to influence it.

Study activities

Orgasmic Meditation (OM)

OM is a structured attention-training practice conducted between two people who follow a predefined set of detailed instructions. One person, the stroker, gently strokes the clitoris of the other person, the strokee, for 15 minutes while both place their attention on the point of contact and notice what they feel. Climax is not the goal. The stroker may be of any gender; the strokee is a woman. Partners check in verbally with each other throughout the session. A more detailed description of OM procedures is provided in Supplementary Material C, reproduced from the published OM container document, Revision 1.5 [21]. Revision 1.4 (2019) was in force during the data collection period; Revision 1.5 (2023) differs from it only in added links to the training application, a renamed section, and one wording change.

Sleep Cycle Assessments

The Sleep Cycle Premium application is available for Apple- and Android-compatible smartphones. It estimates sleep with the device microphone, which records breathing and movement sounds; proprietary machine-learning algorithms, software trained on earlier recordings to recognize patterns, then convert those sounds into estimates of sleep state and likely sleep stage across the night. The variables analyzed here were sleep quality (the primary study outcome), sleep duration, sleep regularity, and time to sleep onset. The application requires a five-night calibration period, during which it adjusts to the user's sounds and movements, and all participants completed this before the 30-day data collection window. Participants placed the device face down on a nightstand with the microphone facing the bed, kept the charger connected, set a preferred wake-up window, started recording when preparing to sleep, and stopped recording on waking.

Consumer sleep applications of this type infer sleep from movement and sound rather than from electroencephalography, the recording of the brain's electrical activity, and validation studies have given mixed results. Fino and colleagues tested four smartphone sleep applications, including Sleep Cycle, against polysomnography, the laboratory recording of brain activity, eye movement, and muscle activity during sleep, and reported that the applications were not sufficiently reliable for sleep-wake detection and performed least well on sleep architecture, the sequence of sleep stages across the night [22]. Bhat and colleagues compared a similar microphone- and movement-based application against in-laboratory polysomnography and reported poor agreement for sleep staging and sleep efficiency, the proportion of time in bed spent asleep, with closer correspondence for total sleep time [23]. Reviews of consumer sleep technology agree that such devices are not interchangeable with polysomnography and that their absolute values should not be interpreted as clinical measurements [24,25].

The research question required 30 consecutive nights of at-home recording from every participant. Polysomnography cannot provide that, and laboratory recording would in any case have altered the sleeping conditions under study. The primary daily OM comparisons reported here compare each participant with themselves, and the same device and algorithm were applied to nights following OM and nights not following OM. A consistent error in the application is therefore largely the same on both sets of nights and is unlikely by itself to produce a difference between them. Random error in the application pushes estimates toward zero and widens confidence intervals. Sleep architecture was not analyzed. The variable the application reports as "sleep quality" is a proprietary composite index, not a validated construct, and is treated throughout as an application-derived index.

Tumescence Survey

The Tumescence Survey is a 15-item self-report instrument written by consultants engaged by the Institute of OM Foundation to capture symptoms and behaviors that practitioners frequently describe as changing with OM practice. It was administered each morning within the daily REDCap questionnaire. Each item is rated on a five-point scale (1 = strong disagreement to 5 = strong agreement). Items 14 and 15 are positively worded and are reverse-scored, so that a 5 counts as 1 and a 1 as 5. The total is the sum of all 15 items after reverse-scoring, with a possible range of 15 to 75. Higher Tumescence Survey scores indicate more psychological distress, dissociation, and craving for addictive substances. No items were excluded, and no missing item responses were estimated; a participant-day was included in survey analyses only if all 15 items were completed. The instrument is provided in Supplementary Material B.

The Tumescence Survey was developed for this line of research and has not been validated against established measures of psychological distress, dissociation, or craving. Its reliability and factor structure were estimated for the first time in this study, in the same sample in which it was administered.

Statistical Analysis

Each participant contributed many nights, so nightly observations are not independent; nights from the same person share that person's stable characteristics, sleep environment, and habitual behavior. Analyses that pool such nights without accounting for which person contributed them understate the standard errors, the uncertainty around each estimate, and mix within-person with between-person variation [12,13]. Associations were therefore estimated with linear mixed-effects models, regression models that give each participant a separate baseline, called a random intercept, so that repeated nights from the same person are treated as related.

For each outcome, a model with no predictors was first estimated to obtain the intraclass correlation, the proportion of the total variation in an outcome that lies between participants rather than between nights within a participant. From the intraclass correlation and the mean number of nights per participant, the design effect (how much the clustering inflates the uncertainty of an estimate) and the effective sample size (the number of independent nights the clustered nights are worth) were derived and are reported alongside each outcome.

The daily OM predictor was split into two parts: each participant's mean rate of practice across the study (the person mean) and each day's deviation from that mean (the person-mean-centered deviation) [12]. The deviation part estimates the within-person association: whether a participant's sleep and Tumescence Survey responses differ on days they practiced compared with their own average. The person-mean part estimates the between-person association: whether participants who practice more frequently differ from those who practice less. Both are reported. Random slopes, which allow the OM association to differ from one participant to the next, were tested and retained where model comparison supported their inclusion.

Models were adjusted for physical exercise on the same day (expressed as each day's deviation from the participant's own mean), day in study, and study site (retreat center versus home). Four sleep outcomes were examined, so p-values for the sleep models are interpreted alongside values corrected by the Benjamini-Hochberg procedure, which adjusts p-values for the number of tests to limit the proportion of false positive findings. Mediation, whether sleep accounts for part of the association between OM practice and next-day scores, was assessed within the multilevel framework. The indirect effect, the part of the association that passes through sleep, was evaluated with a Monte Carlo confidence interval, which is built by repeated random sampling from the estimated coefficients, rather than with the Sobel test, which assumes a normal distribution for that effect [26]. Moderation, whether the association between sleep and next-day scores differs on OM days, was assessed by an OM by sleep interaction term, with the sleep association estimated separately for OM and non-OM days.

Exploratory factor analysis, a method that groups items by how strongly they correlate with one another, was applied to the 15 Tumescence Survey items. The extraction method was principal axis factoring, which models only the variance the items share, with oblique PROMAX rotation, which allows the resulting factors to correlate; factors were interpreted from the pattern matrix, the table of each item's loading on each factor. Factors with eigenvalues ≥ 1.0 (each factor explaining at least as much variance as a single item) were retained, and loading scores were categorized following standard procedure [27]: > .71 as excellent; .63-.71 as very good; .55-.62 as good; .45-.54 as fair; and .32-.44 as poor. Internal consistency, the degree to which the items measure a single thing, was assessed with Cronbach's alpha and with corrected item-total correlations, the correlation of each item with the sum of the other items.

Analyses of individual Tumescence Survey items were not pre-specified and are reported as exploratory, with Benjamini-Hochberg correction applied across the 15 tests. No sample size calculation was performed in advance; the practice occurred naturally in a convenience sample, and enrollment was determined by feasibility. Statistical precision is reported as the effective sample size rather than as power calculated after the fact. All tests were two-sided with alpha set at .05.

Missing Data

For the 36 participants included in data analysis, there were 15 study nights for which all sleep outcome data were zeros. These 15 records were excluded from further analysis as non-recordings. With those exclusions, each of the 36 participants had sleep data for at least 22 nights, and a total of 1,026 nights with Sleep Cycle-assessed sleep measures were included in subsequent analyses. A further 30 nights recorded a total sleep time of zero; these were excluded from analyses of sleep duration and time to sleep onset, which are therefore based on 996 nights. Remaining missing outcome data were handled by maximum likelihood estimation within the mixed-effects models, which use every recorded night from each participant and assume that whether a night is missing does not depend on the unrecorded value once the observed data are taken into account (the missing-at-random assumption).

## Results

Study participants

Forty-one individuals were screened for study participation and were enrolled in the study. One subject was withdrawn for potential concerns over a conflict of interest, one for missing too many assessments, and two were lost to follow-up, leaving 37 participants who completed the study. One participant had data for a single night only and was therefore excluded from further analyses, yielding an analysis set of 36 participants. Twenty-two of the participants included in the analysis set completed the study while sheltered in place during the COVID pandemic at a retreat center in Northern California. The remaining 14 participated from home. Of the 36 participants, 22 were female, and 14 were male. The sample consisted almost entirely of experienced practitioners: 94% had completed more than 100 OMs. Most participants reported no smoking (86%) or alcohol use (97%). Demographics of the study sample are provided in Table 1.

**Table 1:** Participant characteristics for the 36 participants included in the analysis OM: Orgasmic Meditation; SD: standard deviation Values are n (%) unless noted. Ethnicity allowed multiple selections

Characteristic	Category	n	%
Age, years	Mean (SD)	38.8 (8.1)	
Age	18-29	2	6%
	30-39	21	58%
	40-49	10	28%
	50-59	2	6%
	60-69	1	3%
	70+	0	0%
Sex	Male	14	39%
	Female	22	61%
Gender	Male	21	58%
	Female	14	39%
	Other	1	3%
Sexual orientation	Heterosexual	25	69%
	Bisexual	9	25%
	Prefer not to answer	2	6%
Marital status	Single, never married	21	58%
	Married or domestic partner	10	28%
	Divorced	3	8%
	Separated	2	6%
Employment	Employed for wages	23	64%
	Self-employed	10	28%
	Out of work but not currently looking for work	1	3%
	A student	1	3%
	Unable to work	1	3%
Income	$0-$24,999	1	3%
	$25,000-$49,999	16	44%
	$50,000-$74,999	9	25%
	$75,000-$99,999	3	8%
	$100,000 +	5	14%
	Prefer not to answer	2	6%
OM experience	21-50 OMs	1	3%
	51-100 OMs	1	3%
	100+ OMs	34	94%
Smoking	Less than daily	5	14%
	Not at all	31	86%
Education	Some college credit, no degree	2	6%
	Trade/technical/vocational training	2	6%
	Bachelor's degree	19	53%
	Master's degree	9	25%
	Professional or doctoral degree	4	11%
Alcohol use	Yes	1	3%
	No	35	97%
Caffeine use	Yes	20	56%
	No	16	44%
Sleep medication	Yes	1	3%
	No	35	97%
Ethnicity (select all that apply)	American Indian/Alaska Native	1	3%
	Asian	5	14%
	Black/African American	2	6%
	White	33	92%
	Unknown	4	11%
Comorbidities	Depression	1	3%
	Anxiety	2	6%
	Inflammatory disorder	1	3%
	Sleep apnea	2	6%
	Restless leg syndrome	3	8%
	Autoimmune	1	3%
	Menopause	2	6%

Predictor variables: occurrence and frequency of OM practice and physiological climax

Participants who engaged in OM practice during the study period did so with a wide range of frequencies. On the lower end, three participants practiced OM twice during the 30-day study period. At the other extreme, one participant practiced OM at least once on all 30 days. The mean number of days on which participants practiced OM at least once was 14.72, and the median was 14.50. In terms of the total number of OM sessions, two subjects practiced OM twice during the study period, while one participant engaged in 87 OM sessions; the mean number of sessions per participant was 26.53, and the median was 16.50. At least one OM session was reported on 530 of the 1,026 analyzed days.

Most OM sessions conducted during the study did not include a reported climax. Climax was assessed by daily self-report from all participants, irrespective of role. Of the 530 days on which at least one OM session occurred, climax was reported on 103 days (19.4%). Restricted to days on which the participant reported being in the strokee role, climax was reported on 65 of 327 days (19.9%). Across all 1,026 analyzed nights, 197 climax-days were recorded, of which 94 occurred on days without an OM session. Climax counts summed across all study days therefore include climax outside OM practice, so climax is reported within OM days only. Within OM days, among the 26 participants who reported both climax and non-climax days, climax was not associated with sleep quality (b = -0.42, p = .76), sleep duration (+0.8 minutes, p = .92), sleep regularity (p = .83), or Tumescence Factor 2 (p = .91). A nominal association with time to sleep onset (-4.5 minutes, p = .034) was not present when tested across all nights (p = .63).

Sleep and Tumescence Survey Outcome Variables

With night as the unit of observation, across the 1,026 nights recorded during the study, scores for sleep quality (the primary study outcome variable) ranged from 0 to 100, with a mean score of 73.67 (SD: 14.74) and a median of 74.00. Scores for sleep regularity ranged from 0 to 99, with a mean of 84.80 (SD: 11.44) and a median of 88.00. Across the 996 nights with a recorded sleep duration, scores ranged from 35 to 712 minutes, with a mean of 375.18 (SD: 85.95) and a median of 372.50; scores for time to sleep onset ranged from 0 to 180 minutes, with a mean of 20.08 (SD: 20.53) and a median of 10.00. Total scores on the 15-item Tumescence Survey ranged from 15 to 74, with a mean of 33.47 (SD: 12.52).

Tumescence Survey Psychometric Properties

Cronbach's alpha, a measure of how consistently the items measure the same thing, was .92 for the Tumescence Survey, which indicates excellent scale reliability. Corrected item-total correlations ranged from .31 to .78. Only item 15 (I feel I had a refreshing amount of sleep) and item 11 (I feel craving for food, nicotine or alcohol) had correlations below .40 (.31 and .34, respectively).

Exploratory factor analysis of the Tumescence Survey identified two factors with eigenvalues >= 1.0, which together accounted for 52.2% of the variance in the overall scale. Factor 1 included 11 items concerning anxiety, irritability, rumination, negative thoughts, social disconnection, and self-reported relaxation or refreshment. Factor 2 included four items concerning craving, tightness or pain, agitation, and dissociation from the body (Table 2). The factors correlated moderately (r = .59). Because the analysis treated repeated nightly responses as observations, the factor structure is provisional and requires confirmation in a larger independent sample at the participant level.

**Table 2:** Pattern matrix from the exploratory factor analysis of the 15-item Tumescence Survey Values are factor loadings. Reverse-scored items were recoded before analysis.

Item	Factor 1	Factor 2	Assigned
I feel relaxed and open (reverse-scored)	.81	-.05	Factor 1
I feel nervous or anxious	.76	.04	Factor 1
I feel wound up and have a lot on my mind	.76	.05	Factor 1
I feel bottled up	.76	-.09	Factor 1
I have negative thoughts	.75	.08	Factor 1
I feel irritated	.68	.18	Factor 1
I can't stop thinking/I'm in my head	.68	.22	Factor 1
I feel closed off from other people	.68	.15	Factor 1
I feel self-conscious	.65	.15	Factor 1
I had a refreshing amount of sleep (reverse-scored)	.55	-.24	Factor 1
I feel distracted	.44	.39	Factor 1
I feel dissociated from my body	.23	.54	Factor 2
I can't stay still/I feel agitated	.19	.62	Factor 2
I feel tightness or pain in my body	.02	.64	Factor 2
I feel craving for food, nicotine or alcohol	-.36	.95	Factor 2

Clustering of Nightly Observations

Nightly observations clustered substantially within participants (Table 3). With a mean of 28.5 nights per participant, the 1,026 nightly observations were equivalent to between 74 and 187 independent observations, depending on the outcome.

**Table 3:** Clustering and effective sample sizes for the sleep and Tumescence Survey outcomes n: observations; ICC: intraclass correlation coefficient; effective n: observations adjusted for clustering within participants

Outcome	n	ICC	Design effect	Effective n
Sleep quality	1,026	.287	8.9	116
Sleep duration	996	.310	9.5	105
Time to sleep onset	996	.158	5.3	187
Sleep regularity	1,026	.289	8.9	115
Tumescence total	1,026	.381	11.5	89
Tumescence Factor 1	1,026	.354	10.7	96
Tumescence Factor 2	1,026	.466	13.8	74

Associations Between OM Practice and Sleep Outcome Variables

Unadjusted sleep measures on nights following at least one OM session and on nights following days without an OM session are presented in Table 4.

**Table 4:** Unadjusted sleep outcomes following days with and without OM practice OM: Orgasmic Meditation; n: number of observations; SD: standard deviation Values are unadjusted

Measure	Prior day included OM	Nights	Mean	SD
Sleep quality	No	496	76.25	15.14
Sleep quality	Yes	530	71.26	13.94
Sleep duration (minutes)	No	474	395.46	90.54
Sleep duration (minutes)	Yes	522	356.76	77.16
Time to sleep onset (minutes)	No	474	21.72	21.90
Time to sleep onset (minutes)	Yes	522	18.59	19.11
Sleep regularity	No	496	83.64	9.90
Sleep regularity	Yes	530	85.90	12.63

In mixed-effects models that account for the nesting of nights within participants, OM practice was not significantly associated with any sleep outcome (Table 5). Point estimates for sleep quality and sleep duration were in the direction of poorer and shorter sleep following days that included OM. Confidence intervals included zero in every case, and no outcome approached significance after correction for four comparisons. Between-person associations were likewise nonsignificant (sleep quality b = 4.56; p = 0.404; sleep duration b = 3.45; p = 0.910; time to sleep onset b = 0.02; p = 0.998; sleep regularity b = 8.67, p = .078).

**Table 5:** Within-person associations between OM practice and sleep outcomes on the following night OM: Orgasmic Meditation; b: regression coefficient; CI: confidence interval; p: probability value Negative values indicate lower outcomes following OM

Outcome	Within-person b	95% CI	p
Sleep quality (index points)	-1.87	-3.96 to 0.21	.079
Sleep duration (minutes)	-10.11	-22.37 to 2.15	.106
Time to sleep onset (minutes)	+2.01	-1.24 to 5.26	.225
Sleep regularity (index points)	+0.05	-1.57 to 1.68	.948

Covariate adjustment changed these estimates little; in models that added covariates one at a time, the within-person coefficient for sleep quality moved from -1.96 unadjusted to -1.87 fully adjusted. The absence of significant associations therefore reflects the modeling of the nested data rather than covariate adjustment. Study site, in contrast, was strongly associated with sleep: participants at the retreat center recorded sleep quality 11.66 index points lower (p = .002) and slept 68.3 minutes less per night (p = .002) than participants at home.

Individual participants differed in the direction of association. Among the 32 participants contributing both OM and non-OM nights, 18 recorded poorer sleep quality following OM days and 14 recorded better, with per-participant differences ranging from 17.3 index points worse to 13.5 points better (SD of per-participant differences = 7.1).

Associations Between OM Practice and Tumescence Survey Scores

Tumescence Survey Factor 2 scores were lower on mornings following a day that included at least one OM session (Table 6). Lower Factor 2 scores reflect lower combined ratings across craving, tightness or pain, agitation, and dissociation. The total-score estimate narrowly missed statistical significance (b = -1.65; 95% CI: -3.31 to 0.003; p = .0504), while Factor 1 was not associated with OM practice.

**Table 6:** Within-person associations between OM practice and next-morning Tumescence Survey scores b: regression coefficient; CI: confidence interval; p: probability value; d: standardized effect estimate Negative values indicate lower scores following OM

Outcome	Within-person b	95% CI	p	d
Tumescence total (15 items)	-1.65	-3.31 to 0.00	.050	-0.13
Factor 1 (anxiety, rumination)	-0.88	-2.25 to 0.49	.208	-0.09
Factor 2 (somatic, craving)	-0.77	-1.17 to -0.37	< .001	-0.24

When OM practice was entered as the number of sessions rather than as a yes or no indicator, each session on a given day was associated with a further reduction of 0.27 points (p = 0.009). This coefficient does not describe a graded response to repeated sessions. When the occurrence of practice and the number of sessions were entered together, the association depended on whether the participant practiced at all that day (b = -0.79; p = 0.007), and each session beyond the first added nothing detectable (b = +0.01; p = .93). The association was unchanged when the three highest-frequency practitioners were excluded (b = -0.80; p < .001). When the OM slope was allowed to vary across participants, the estimate decreased to b = -0.67 (p = .056), which indicates that the association is not uniform across individuals. The size of the association did not depend on how often a participant practiced (OM by frequency interaction, p = 0.47). There was also no evidence of accumulation across the study: sleep quality, the total score, and Factor 2 showed no linear trend across the 30 days (p = 0.77, 0.16, and 0.83) and no association with cumulative sessions to date (p = 0.69, 0.36, and 0.66).

Exploratory Item-Level Analysis of Factor 2

Each of the 15 Tumescence Survey items was modeled separately. These analyses were not pre-specified and are reported as exploratory. Two items remained significant after Benjamini-Hochberg correction across the 15 tests: dissociation from the body (b = -0.32; q < .001; d = -0.30) and agitation (b = -0.23; q = .022; d = -0.21). The craving item (b = -0.08; q = .470) and irritability item (b = -0.03; q = .939) were not associated with OM practice. The remaining items also did not survive correction (Table 7). Thus, the item-level evidence is specific to dissociation and agitation and does not support a claim that OM reduced craving or irritability in this sample.

**Table 7:** Exploratory item-level associations between OM practice and next-morning Tumescence Survey responses OM: Orgasmic Meditation; b: regression coefficient; p: unadjusted probability value; q: corrected probability value; d: standardized effect estimate Negative values indicate lower scores following OM

Item (higher score = greater distress)	b	p	q	d
I feel dissociated from my body	-0.322	<0.001	<0.001	-0.30
I can’t stay still/I feel agitated	-0.233	0.003	0.022	-0.21
I can’t stop thinking/I’m in my head	-0.198	0.025	0.078	-0.16
I feel closed off from other people	-0.197	0.017	0.078	-0.17
I feel distracted	-0.191	0.026	0.078	-0.15
I feel relaxed and open (reverse-scored)	-0.163	0.067	0.167	-0.14
I feel tightness or pain in my body	-0.134	0.131	0.281	-0.10
I feel self-conscious	-0.096	0.297	0.495	-0.07
I feel craving for food, nicotine or alcohol	-0.083	0.251	0.470	-0.08
Remaining six items	≤0.04	>0.70	>0.93	~0

Associations Between Tumescence Survey Outcomes and Sleep Variables

The association between sleep duration and Factor 2 differed in sign according to the level of analysis (Table 8). Within participants, longer sleep on a given night was associated with lower Factor 2 scores on the following morning. Between participants, those with longer average sleep reported higher Factor 2 scores. An analysis that does not separate the two levels returns a positive association, which reflects the between-person pattern. The same reversal was observed with sleep quality in place of sleep duration (within-person b = -0.017; p = .006; between-person b = +0.108; p = .014). The between-person estimates in Table 8 are not adjusted for study site. Twenty-two of the 36 participants completed the study at the retreat center, and site was strongly associated with sleep, so adjustment for site reduces the between-person estimates (sleep duration b = +0.86; p = .137; sleep quality b = +0.100; p = .060). The within-person estimates are unchanged by that adjustment, so the difference in sign between levels does not depend on it.

**Table 8:** Within-person and between-person associations between sleep duration and next-morning Tumescence Survey Factor 2 scores ρ: pooled correlation; b: regression coefficient per additional hour of sleep; p: probability value Higher Factor 2 scores indicate greater reported symptoms

Sleep duration and Tumescence Factor 2	Estimate	p
Pooled, levels not distinguished	rho = +0.143	-
Within-person (per additional hour)	-0.20	0.002
Between-person (per additional hour)	+1.05	0.016

Mediation and Moderation by Sleep

Multilevel mediation analysis tested whether sleep accounted for any portion of the association between OM practice and Factor 2. It did not. The path from OM practice to sleep duration was nonsignificant (b = -10.11; p = .106). The path from sleep duration to Factor 2 was significant (b = -0.0037; p < .001), but the indirect effect, the part of the association that passes through sleep, was not significant (Monte Carlo 95% CI: -0.007 to 0.096), and the direct association between OM practice and Factor 2 was essentially unchanged with sleep duration in the model (b = -0.85; p < .001).

We also examined whether the association between sleep and next-day scores differed on OM days, which would indicate that sleep contributed less on days when participants practiced. The OM by sleep interaction was non-significant for Factor 2 (p = .854) and for the total score (p = .872), and the association between an additional hour of sleep and Factor 2 was comparable on OM days (b = -0.17; p = .034) and non-OM days (b = -0.24; p = .015). Entered together, OM practice and sleep duration were independently associated with Factor 2 in the same direction: each additional hour of sleep was associated with a 0.22-point lower score the next morning, and a day of OM with a 0.85-point lower score. The ratio of the two coefficients gives the amount of additional sleep associated with the same change as a day of OM: 3.8 hours for Factor 2 (95% CI: 1.8 to 9.5) and 1.7 hours for the total score (95% CI: 0.2 to 3.9). For the total score, the OM coefficient with sleep duration in the model was -1.95 (95% CI: -3.64 to -0.26; p = .024), compared with -1.65 (p = .0504) without it. When this sleep-adjusted association was allowed to vary among participants, the estimate weakened and was no longer statistically significant (b = -1.56; 95% CI: -4.30 to 1.17; p = .262). For scale, a participant’s own sleep varied from night to night by 1.2 hours, and 90% of nights fell within 1.7 hours of that participant’s mean.

Checks of Temporal Selection and Recording Window

Because participants chose when to practice, prior-night sleep could have influenced whether they practiced the following day. Within participants, each additional hour of prior-night sleep was associated with lower odds of practicing the next day (odds ratio: 0.83; p = .014), and each 10-point increase in prior-night sleep quality was associated with lower odds of practicing (odds ratio: 0.85; p = .008). Prior-night sleep did not predict the next night's sleep duration or quality, and adjustment for prior-night sleep left the OM estimates materially unchanged. These analyses reduce but do not eliminate the possibility that unmeasured daily circumstances influenced both the decision to practice and the following outcomes.

OM practice was not associated with a later bedtime (b = +2.58 minutes; p = .745), shorter time in bed (b = -9.29 minutes; p = .129), or lower sleep efficiency (b = -0.52 percentage points; p = .398). Holding bedtime constant produced a similar estimate for sleep duration (b = -9.69 minutes; p = .095). The data therefore do not support a simple explanation in which the 15-minute session delayed bedtime and shortened the recorded sleep window, although the time of each OM session was not recorded.

## Discussion

No previous study has examined the relationship between OM and app-assessed sleep. Across 1,026 nights contributed by 36 experienced practitioners, OM practice was not associated with sleep quality, sleep duration, time to sleep onset, or sleep regularity once the nesting of nights within participants was modeled. OM practice was associated with a small reduction in Factor 2 of the Tumescence Survey, driven at the item level by lower ratings of dissociation from the body and agitation.

An analysis that treats nightly observations as independent yields significant associations on all four sleep measures in these same data. The difference has two sources, and both move the pooled estimate in the same direction. Participants who practice OM more frequently are different individuals from those who practice less, and in this sample they also differed by study site. An analysis that does not separate levels therefore attributes to the practice differences that may belong to the practitioner. Restricting the comparison to within participants reduces the sleep quality difference from approximately five index points to approximately two. Repeated observations from the same individual are also correlated, and the intraclass correlations in Table 3 show that 1,026 nights are equivalent to substantially fewer independent observations, which widens the confidence intervals. Participants also differed from one another in direction: 18 recorded poorer sleep quality following OM and 14 recorded better, a range of more than 30 index points. Any relationship between OM practice and sleep in this sample is therefore not uniform across individuals.

The absence of a detectable association is not a demonstration that none exists. With effective sample sizes between 105 and 187 for the sleep outcomes, the study could not detect small effects, and the confidence intervals in Table 5 remain compatible with modest reductions in sleep quality and duration. They do provide the precision estimates needed to design a study that could answer the question.

The association between OM practice and Factor 2 of the Tumescence Survey remained after the correction for repeated nights and after exclusion of the three most frequent practitioners. When the association was allowed to vary across participants, however, the estimate weakened to p = .056, which suggests meaningful individual variation. At the item level, participants rated two statements lower on mornings after OM, "I feel dissociated from my body" and "I can’t stay still/I feel agitated," and only these two remained associated with OM after correction for multiple comparisons. These two items correspond to bodily presence and restlessness, capacities that the practice may train through sustained attention to sensation. The observational design cannot determine whether the practice produced these changes, and expectancy, self-selection, daily circumstances, or other unmeasured factors remain possible explanations.

The craving item had the highest loading on Factor 2 and therefore influenced the interpretation of that factor. It was not associated with OM practice, and removing it from the factor left the composite association essentially unchanged. A factor label describes which items vary together, not which items respond to an exposure. These data support an association with less dissociation and agitation, but not with craving for food, nicotine, or alcohol or with irritability.

The relationship between sleep and Factor 2 also depended on the level of analysis, and the sign reversed between levels. Within participants, longer sleep predicted lower next-morning Factor 2 scores, consistent with conventional expectation. Between participants, those who habitually slept longer reported higher Factor 2 scores. A pooled analysis returns the between-person pattern and could be read as evidence that longer sleep accompanies greater somatic discomfort and disconnection. Kalmbach et al. [9] reported the same reversal.

Participants reported lower total Tumescence Survey scores on mornings after OM, although this association narrowly missed statistical significance in the primary model (p = 0.0504). When sleep duration was included in the model, so that OM and non-OM mornings were compared after equivalent amounts of sleep, total scores after OM were significantly lower (b = -1.95; 95% CI: -3.64 to -0.26, p = 0.024), and the Factor 2 association was unchanged. When the sleep-adjusted association was allowed to vary among participants, it weakened and was no longer statistically significant (b = -1.56; 95% CI: -4.30 to 1.17; p = .262), as the Factor 2 association did. The lower scores after OM were therefore not explained by differences in sleep: OM practice was not reliably associated with sleep, sleep did not mediate the Factor 2 association, and the association between sleep and next-day scores did not differ between OM and non-OM days. The difference associated with a day of OM was the same size as the difference associated with 1.7 additional hours of sleep for the total score and 3.8 additional hours for Factor 2. Murnieks and colleagues used this comparison to express the size of a mindfulness effect in hours of sleep [28]. The comparison describes the relative size of two associations from one observational model. It does not show that OM replaces sleep or reduces sleep need.

Earlier studies provide a limited basis for asking whether contemplative practice changes daytime functioning independently of sleep. Kaul and colleagues found that 40 minutes of meditation improved reaction time in ten novice meditators, whereas a 40-minute nap did not, and seven long-term meditators slept about 2.6 hours less per night than 23 matched controls [29]. Murnieks and colleagues found that about 10 minutes of daily mindfulness practice was associated with the same reduction in exhaustion as an extra 44 minutes of sleep per night [28]. A meta-analysis of 18 randomized trials found that mindfulness improved sleep quality relative to nonspecific controls but not relative to established sleep treatments [11]. These studies examine different practices, populations, and outcomes. They justify direct measurement of alertness and performance after OM.

Most OM sessions in this study did not end in reported climax. Climax was reported on 103 of 530 OM days (19.4%), and within OM days it was not associated with sleep quality, duration, regularity, or Factor 2. Research on sexual activity and sleep has largely examined perceptions, satisfaction, frequency, or orgasm rather than a standardized genital attentional practice. A systematic review of tactile intimacy found limited evidence linking sexual function, sexual satisfaction, or masturbation with sleep quality, no evidence for an association with sexual frequency, and more positive findings in subjective than objective studies [10]. OM therefore provides a useful way to separate genital stimulation and partnered attention from climax. This study was not designed to estimate the causal contribution of any one component, but it shows that OM and climax were distinct events on most practice days.

Limitations

The most important limitation is the sample. Participants were recruited through the OneTaste mailing list and from a retreat center; eligibility required weekly practice, and 94% reported more than 100 lifetime OM sessions. There was no comparison group. Experienced practitioners likely expect the practice to help them, and that expectation may have influenced their morning responses. The findings apply to experienced practitioners and should not be extended to OM-naive individuals or the general population. The sample was also predominantly White and highly educated.

The Tumescence Survey has not been validated against established measures of distress, dissociation, agitation, or craving, and its factor structure was derived in the same sample in which it was administered. The exploratory factor analysis also used repeated nightly responses without explicitly modeling their clustering, so the apparent sample size overstates the independent information available for determining the factor structure. A lower score on this survey is not the same as improved well-being. The survey was completed the morning after practice, so the associations concern next-day status rather than an immediate response. The item-level analyses were not planned and require replication with validated measures.

Sleep was estimated by a consumer application that has not been validated against polysomnography or laboratory sleep recording for this use, and its sleep quality score is a proprietary index. The primary daily OM comparisons in this study were within-person, with the same device and algorithm applied to OM nights and non-OM nights, so a consistent error in the application cannot produce a difference between the two. The application infers sleep from sound and movement at the bedside. A partner may be more likely to be present on nights after practice, and partner movement could raise the estimated wakefulness on those nights. This possible error cannot be excluded with these data. A study that pairs the application with laboratory sleep recording on a subset of nights would test it.

Thirty-six participants contributed 1,026 nights. The effective sample size, the number of independent observations the clustered nights are worth, ranges from 74 to 187 (Table 3), and conclusions generalize across participants, not across nights. With 36 participants, the study can detect only moderate to large between-person effects, and every between-person finding is exploratory. A study powered at the participant level would need to enroll several times as many people.

Twenty-two of the 36 participants completed the study while sheltering in place together at a retreat center during the COVID-19 pandemic. Site was strongly associated with sleep quality and sleep duration, and all 15 participants who practiced on 20 or more days were at the retreat (correlation between frequency and site, 0.77). Apparent differences by practice frequency reversed sign once site was adjusted for: sleep quality -4.04 index points (p = 0.149) became +3.77 (p = 0.243), and sleep duration -32.9 minutes (p = 0.052) became +18.4 minutes (p = 0.301). Site and practice frequency cannot be separated in these data, so the study cannot say whether people who practice more differ from people who practice less. Sex and practice role are also completely confounded, so that neither can be examined apart from the other: all 22 women were stroke survivors, and all 14 men were strokers, and only two participants ever recorded both roles, so no analysis by sex is reported. A study that recruits from several sites and includes women in the stroker role could separate these.

Enrollment in a study about OM may itself have changed behavior. Daily reporting of practice may have increased its frequency, and daily self-monitoring of sleep and Tumescence Survey responses may have influenced the responses. This reactivity, the behavior change that comes from being observed, is inherent to daily diary designs and cannot be separated from the associations reported.

The earlier OM studies share authors and funding with this one. This study was funded by the Institute of OM Foundation, which engaged the author as a consultant. The de-identified dataset is publicly available (OSF, DOI 10.17605/OSF.IO/8TKFW). Independent replication of any OM finding remains important. The study was registered before recruitment, approved by an independent review board, and collected 30 consecutive nights from every participant, so each person served as their own control.

## Conclusions

In this 30-day observational study of experienced practitioners, OM practice was not associated with app-assessed sleep quality, duration, time to sleep onset, or regularity once the nesting of nightly observations within participants was modeled. Point estimates for sleep quality and duration were directionally consistent with poorer and shorter sleep, but confidence intervals included zero, and individual participants differed in direction. These data neither demonstrate nor exclude a relationship between OM practice and sleep.

The primary model associated OM practice with a small reduction in Tumescence Survey Factor 2 scores on the following morning, although the confidence interval included zero when the OM association was allowed to vary among participants. In exploratory item-level analyses, dissociation from the body and agitation remained associated with OM after correction for multiple comparisons. Craving and irritability did not. When sleep duration was taken into account, participants also reported significantly lower total Tumescence Survey scores on mornings after OM, and the lower scores were not explained by differences in sleep; this estimate too weakened when the OM association was allowed to vary among participants. The difference associated with a day of OM was the same size as the difference associated with 1.7 additional hours of sleep for the total score and 3.8 additional hours for Factor 2. The observational design cannot establish that OM caused the difference.

Climax was reported on approximately one in five OM days and was not associated with the sleep outcomes within OM days. OM and climax were therefore separate on most practice days in this sample. This distinction makes OM a useful model for future studies that compare the contributions of genital stimulation, partnered attention, contemplative practice, and climax.

These findings are observational, drawn from a small, highly selected sample using a consumer sleep application and an unvalidated survey, and do not establish causation. A controlled study should randomize the timing of practice; include OM-naive participants; use validated measures of dissociation, agitation, craving, irritability, sleepiness, vigilance, and functioning; and pair at-home measurement with laboratory sleep assessment. An active comparison condition using partnered nongenital touch or solo attention would help separate the components of OM. Only a study that measures performance and sleep pressure after controlled sleep opportunities can test whether OM changes how much sleep a person needs.

## Appendices

Supplemental material A: demographics survey

A demographics survey was administered to participants and captured age, sex, gender, ethnicity, race, highest level of education attained, annual household income, employment status, marital status, lifetime OM experience, and whether the participant regularly used a sleep medication.

Supplemental material B: Tumescence Survey

The Tumescence Survey is a 15-item self-report instrument. Each item is rated on a 5-point Likert scale (1 = strong disagreement, 2 = mild disagreement, 3 = neither agree nor disagree, 4 = mild agreement, 5 = strong agreement). Respondents indicated the degree to which they agreed with each of the following statements: (1) I feel nervous / anxious; (2) I feel wound up and have a lot on my mind; (3) I feel tightness or pain in my body; (4) I feel irritated; (5) I feel self-conscious; (6) I can't stop thinking / I'm really in my head; (7) I can't feel my body; (8) I feel closed; (9) I feel bottled up or like I could cry; (10) I can't stay still / I feel agitated; (11) I feel craving for food, nicotine, or alcohol; (12) I feel distracted; (13) I can't stop thinking negative thoughts; (14) I feel relaxed and open; and (15) I feel I had a refreshing amount of sleep. Items 14 and 15 are reverse-scored when computing the total score.

Supplemental material C: the container and form of orgasmic meditation

Reproduced from the Institute of OM container document, Revision 1.5 (2023). Revision 1.4 (2019) was in force during the data collection period; Revision 1.5 differs from it only in added links to the training application, a renamed section, and one wording change.

Foreword

This document is a supplement to basic training in the practice of Orgasmic Meditation® (OM) delivered through current or past training options such as online training materials, an introductory course taught by an OM Life® Certified Teacher, or private training offered by an OM Life® Certified Coach. It is not a replacement for any of those things. The Institute of OM™ strongly suggests receiving some form of basic training through one of the aforementioned resources before attempting to practice. The Orgasmic Meditation® Container has been through a number of different (mostly incomplete) iterations before the creation of this document. You’ll find them in the book, Slow Sex: The Art and Craft of the Female Orgasm (Grand Central/Hachette, May 2011), by Nicole Daedone, and in the now retired “OM Essentials” and “How to OM” digital documents given to OneTaste® course participants after having been trained in courses in years past. Each of those documents represents both a version and a corresponding angle on the practice of OM and was “just the right thing” for the time and purpose they were meant to fulfill. The version in the book is written for a mainstream audience of existing couples practicing alone (likely never having access to OneTaste® or The Institute of OM™ training), where the other documents reflect a more nascent, close-knit community practice where the materials didn’t have to be comprehensive because new practitioners would “catch on” to all the nuance through exposure to experts. The purpose of this document is to meet the practice where it is in the present time: A modern and innovative concept that is becoming mainstream, and has been shown to increase connection, empathy, and generosity, and forge deeper connections with others. In precisely the same way that the common internet communication language that allows our smartphones, computers, cars, refrigerators, etc. to seamlessly interconnect had to be standardized at the birth of the Internet, Orgasmic Meditation® must also be well documented and standardized so that no matter where a practitioner may go or who they OM with, OM is OM. The distinctions contained in this document are crucial to understanding that Orgasmic Meditation® is a specific practice performed under detailed instructions and compliance guidelines, not a sex act. There will be revisions to this document listed at the end in a “revision history” section that lists the changes as each progressive version improves upon the last. Please send suggestions to container@instituteofom.com.

The Container vs The Form

The “container” of the practice refers to the unchanging, nonnegotiable aspects of the practice which include the following:

• No reciprocation-both roles in OM are done solely for their own enjoyment, not for any additional benefit or expectation of compensation

• 15-minute duration

• Safeporting at two distinct points

• Stroker remains fully clothed; strokee only undresses from the waist down

• “Nothing extra” in the nest, i.e., no romantic gestures, added or subtracted steps, changes to the time limit, etc.

Think of the container as both the context and the environment created to hold the practice. The “form” of the practice refers to how practitioners conduct themselves in and around the practice, i.e., how we do what we do inside the container in terms of intention, behavior, and technique. When we OM, each partner overtly opts in to holding the container and practicing good form. Falling out of the form is not always the same as breaking the container, though both matter greatly to the integrity of the practice. The form of OM serves us by showing us the gap between where we are now and our aims so that we may examine what lies in the expanse and continue to refine our practice with rigor. Falling out of the form due to habit and conditioning is human and expected to happen from time to time. If we could all maintain perfect uninterrupted attention while staying connected to our bodies in the simplest of ways, our practice wouldn’t be nearly as rich or necessary! Both container breaking and bad form can be deliberate or accidental. Rigor is a necessary element in any OM practice. A few examples:

- OMing with the goal of achieving climax out of habit (on behalf of either partner) is a common example of behavior that is not breaking the container yet would usually be considered an accidental lapse in form.

- If your timer battery died mid-OM and you went longer than 15 minutes, that would be an accidental container break.

- An example of good form would be avoiding towel contact with the anus during the towel stroke.

- In contrast, wittingly allowing the timer to go over (or under) 15 minutes, hitting on your partner during the execution of the practice, charging money or accepting compensation for OMs, etc., would break the container deliberately. Deliberate or accidental, each practitioner is responsible for their actions in and education surrounding the container. Because of our cultural bias towards climax, the term “goal-lessness,” to the equal intrigue and appeal of many who find OM, often starts out as being synonymous with “not climax-centric,” or “not created to produce climax”. Indeed, people seem to know that there is more to connection and orgasm than what’s been prescribed as the one-size-fits-all approach. As strokers and strokees begin their practice, they often confront their relationship to climax as they feel their way through their experiences, noticing their attachments to various feelings, stories, and expectations of how things “should go.” The attachment to climax is perhaps the most obvious preoccupation reported by practitioners, but while it's perhaps the most obvious, it's far from the only one. In this context, being “goal-less” is more closely defined as not being habituated to any specific experience of orgasm. For example, there are some who shy away from climax and instead prefer stillness and calm. There are those who like a sense of play and those who prefer a sexier frequency of orgasm. Orgasm includes all channels, and if you OM, you are by definition asking to be moved by your body’s own electricity into states that might feel unfamiliar. The principle of “goallessness” isn’t meant as an instruction to relinquish all aims or desires to receive benefits from the practice. On the contrary, OM is designed as a “goalless” practice in order to increase our receptivity to its benefits. Releasing our experience from the bondage of a fixed goal, outcome, or flavor opens the possibility for the unplanned and unexpected to occur. The beauty of the practice is that if we agree to let go of what we think orgasm should feel like and rather learn to be with the beauty of it exactly as it is. Training in the practice of OM (in person, via video conference, or online) is designed to advise practitioners on good form and container keeping! Contact info@instituteofom.com for more details.

The Menstrual Cycle and Personal Care

Our culture has many expectations and prescriptions for how a strokee should care for and present their genitals as well as the surrounding areas. Strokees may feel as though they must somehow prepare their genitals for the benefit of their stroker. OM does not require any specific form of preparation beyond that of a person practicing their own best sense of personal care, whatever that means to them. Many strokees have reported that OMing on their cycle is beneficial in a variety of ways. OMing on your menstrual cycle is a personal choice for the strokee, as is informing your partner that you are on your cycle. Lastly, bear in mind that for many, OM is practiced as part of a process to reclaim their humanity. Every practitioner will be at different places in their practice, doing their own personal best to embrace their own and others’ humanity. Should either partner get their feelings hurt, as with most aspects of this practice, it’s often wise not to take it personally.

OM Training, Practice, and Practice Forms

The online training platform created by OneTaste® and OM Life® Certified Coaching in the practice of Orgasmic Meditation® helps practitioners learn the practice and expand the range of what's possible in their Orgasmic Meditation® experience through personal training and instruction in the practice of OM. From time to time, a client may be offered a certain focus for an OM or series of OMs in order to help the client work on some aspect of themselves through the practice. This is referred to as an OM Practice Form. For example, if a client is having trouble speaking their desires, the trainer may offer a custom OM Practice Form where the client uses their voice in a way that helps them with their issue but would be outside the normal form of offers and requests found in a typical OM. In cases like this, the modified form should not be thought of as a lapse. Other examples may include OMs where one partner or another speaks aloud beyond offers and requests or strokes with a specific intention, i.e., purely downstrokes for the duration of the OM. OM Practice Forms may change the form of the practice but do not modify the container, i.e., 15 minutes, no commerce, finger-to-clitoris only, etc.

Before the Nest

Standard training in Orgasmic Meditation® is available through the following channels:

1. Online using the iOS app, ‘OM App: Partnered Meditation’ found here (or the web app version compatible with Android phones and desktop computers).

2. Private coaching offered by an OM Life® Certified Coach in good standing (or certification candidate). Note: OM Life® Certified Coaches will never be present with clients while clients are OMing. All training is done verbally and/or via fully clothed demonstration either in person or via telecommunication. After an exhaustive amount of informal research and feedback on the topic, we suggest that potential OM partners receive training from official sources. We suggest asking your potential OM Partners where and how they were trained to find out who has been trained. Asking potential partners if they have read this document comprehensively is also recommended.

Asking Form

In OM, we ask in a way that reflects the reality that you’re doing something together, not that one partner is perhaps doing something to or for the other. Additionally, when asking somebody to OM, keep the request simple. Mixing invitations to OM with other offers like dates, sex acts, and miscellaneous social activities is highly discouraged. This helps eliminate the expectation of commerce from the practice and otherwise keeps OMing relationships clean between practitioners. One of OM’s greatest benefits is that it is based on simplicity, nothing more or less than needed, and it need not rely on any other activities or forms of relationship in order to exist. You’ll want to set a specific time and place for the OM rather than leaving those details undefined. Either the stroker or the strokee may ask for the OM. OM is desire-based, so one partner must express desire to OM. The person receiving the request is free to accept or decline without explanation. People say no for many reasons that aren’t personal. If you get a “no”, simply say “thank you” and remember that it might just be “no” for right now. Proper form: Would you like to have an OM? Would you like to OM? Improper form: Will you stroke me? Can I OM you? We should OM sometime!

Container and Form in the Nest

At minimum, a nest consists of a fresh washcloth, at least three pillows (head, and one for each leg), a blanket, gloves, lube, a timer, and a firm pillow for the stroker to sit on. It’s recommended but not mandatory to place a yoga mat under the blanket if OMing on a hard floor. The following description is the practiced, non-negotiable series of events that comprise the experience of Orgasmic Meditation®.

1. Get into position in the following way: Strokee takes off their pants and lies down in the nest. Stroker removes their footwear, and optionally their belt, and besides those articles remains fully clothed. Once the strokee is in the nest, the stroker mindfully enters the nest by stepping over and across the strokee with their left foot and sits down next to the strokee on the strokee’s right-hand side so that the stroker may stroke with their left index finger. Form Note: Strokers might not be able to distribute their weight as described above and can modify the entry into the nest to something suitable to their physical condition, such as first sitting down on the sitting cushion facing the foot of the nest and then swiveling to face the strokee.

2. Once both partners are physically comfortable, the stroker gives the first of two safeports, informing the strokee that they are about to give some grounding pressure. Example: I am going to give you some grounding pressure now. During the grounding pressure, the palms of the stroker’s hands press down on the insides of the strokee’s thighs, one hand per thigh, using medium pressure. This step is not a massage, nor is it a light “lover’s touch”. The hands remain relatively flat, surface to surface against the thighs. This step typically lasts between 10-15 seconds at most.

3. The noticing step consists of a one- or two-sentence value-neutral (in terms of shape, color, location, texture, etc.) physical description of some aspect of the strokee’s genitals. The etiquette is simply for the strokee to acknowledge this observation by saying “thank you” afterwards. Example: Your inner labia are a light coral color and bend slightly to the left”. This step is brief, around 10 seconds. During the noticing step, metaphor, desires, compliments, and the like are not part of the practice. Stroker keeps their hands on the strokee’s thighs during the noticing step!

4. Stroker puts on gloves, taking care that the glove is flat and wrinkle-free as it adheres to the left index finger. Form Note: It never hurts to keep the elbow or forearm connected to the strokee’s thigh to maintain physical connection during this step.

5. Lube is applied to the stroker’s gloved left index finger and gloved right thumb.

6. Stroker gives the second of two safeports, letting the strokee know they are about to touch their genitals. Example: I’m going to touch your genitals now. Form Note: Safeporting happens at 2 specific times - grounding pressure and genital contact, as well as if either partner needs to make some kind of physical adjustment (postural or otherwise) during the OM. Other safeports such as stepping over the strokee are extraneous and should not be included or expected. An example of an appropriate safeport during an OM might be if the strokee gets a leg cramp and needs to shift positions, or if the stroker needs to remove their hands from the strokee’s genitals to make some other postural adjustment. Safeporting before normal bodily functions (flatulence, coughing, sneezing) is optional.

7. Stroker begins the 15-minute countdown before genital contact is made (in an OM circle, it begins when the Circle Holder indicates that time has begun). A timing device is used to keep time.

8. Stroker carefully makes contact with the strokee’s genitals using the following form known as the lube stroke:

(a) Form A: With the labia gently parted using the middle finger and thumb of the left hand, the left index finger begins at the base of the introitus and deliberately, with one stroke, draws the tip of the index finger up through the parted labia and onto the clitoris. Form B: With the labia gently parted using the middle and ring finger of each hand, the left index finger begins at the base of the introitus and deliberately, with one stroke, draws the tip of the index finger up through the parted labia and onto the clitoris. (b) At this point, the left thumb is placed at the clitoral hood and gently pulls the hood back to expose the glans of the clitoris. (c) The right thumb is placed at the base of the introitus: not penetrating or moving, simply resting stationary with no more than the middle of the thumbnail over the threshold of the introitus. It simply rests with solid contact at the base of the introitus to provide a constant ground and feel contractions. (d) Lube stroke form notes:

1. The two forms of lube stroke offer a set of practical alternatives to difficult anatomical (both genital and body shape/size/ability) combinations. Pick the one that best suits your use case.

2. Depending on what form is used, the thumb may be placed at the base of the introitus before or after the labia are parted, and the thumb and index finger of the left hand are settled at the hood and clitoris, respectively.

3. There are from time to time physical size and flexibility issues that prevent strokers from being able to place the right thumb at the base of the introitus. In such cases, it may be placed on the strokee’s right thigh or disregarded. Strokees may also request to omit the thumb due to pain or sensitivity in the introitus. Please refer to an OM Life® Certified Coach for guidance if desired.

4. Due to contractions and other naturally occurring motion during the OM, the right thumb may either recede or advance in the introitus. Strokees can simply ask for the adjustment: “Can you please move your thumb further out/ in my introitus?” Should a stroker notice, good form dictates that they return to the proper placement.

9. During the OM, offers and requests can be made. Offers and requests exist in order to help practitioners find the spot of highest sensation between them, not to satisfy one partner’s agenda at the expense of the other’s. It’s good form to assume your partner is offering or requesting in order to find the most resonant connection between the two of you. Examples of proper form for requests and offers: Can I have a firmer stroke? Specific and actionable. After the stroker hears and answers the request, the typical form is for them to say “thank you”. Examples of the form of an offer: Would you like a slower stroke? Specific and simply yes/no.

10. Once 13 minutes have elapsed, the stroker gives a 2-minute warning. “2 minutes” is always vocalized by the stroker (or Circle Holder). Downstrokes in these final two minutes (and for the rest of the OM) remain focused on the clitoris and employ the left index finger only for stroking. Both partners set their intention to come down. Form Note: The left thumb remains in contact with the strokee’s clitoral hood/lower pelvic area during the final two minutes. The thumb does not float above the hood.

11. Once time runs out, the stroker places their left hand flat against the strokee’s genitals with fingers pointed downwards. There is no gap between fingers, and the aim is for both sets of labia to be enclosed by the left hand of the stroker. The heel of the right hand, fingers pointed up, is used to press against the back of the left hand. Both hands are used in conjunction to apply firm, sustained pressure towards the strokee’s head and/or down on the strokee’s pubic bone. This step typically lasts between 10-15 seconds at most.

12. The towel stroke is done, and then the gloves come off. The form is to do the towel stroke first and then remove the gloves to provide the most sanitary environment possible.

13. One frame is shared per partner after the strokee has sat up. Frames are specific, low abstraction, and typically last 10-15 seconds. Frames include one or some of the following descriptors of a bodily sensation in terms of shape, temperature, vibration, location, movement, etc., and may also include a description of when in the OM the frame occurred. Frames involving metaphor, imagery, intentions, desires, or abstract concepts aren’t generally considered frames. Good form: There was a moment I felt a light buzzing warmth in my chest. Poor form: There was a moment I felt connected and like we were flying and I really wanted to kiss.

14. Exit the nest and strokee puts their pants back on. Put the nest supplies away, and the OM is complete! You’re all done!

The Container Is Key

In order for us to enter into a state of orgasm, we must feel safe. This is especially true for women. Why? Because her internal guard dog, the center of vigilance, is about to go off duty for a while. Orgasm is, by definition, letting ourselves open up to what we normally might not be feeling. For many, this is akin to letting go of control. The consistent structure of OM creates a sense of regularity that our bodies learn to trust over time, allowing us to increasingly let our guard down and open up to what we’re feeling. Sticking to the structure, for example, ending the OM at the agreed-upon time, even if it still feels good, gives us something extremely valuable in the long term: a practice that we can rely on for both the orgasm and limbic connection we hunger for. The integrity of the container is like the foundation of a house you live in: everything that happens either builds it up or tears it down. On a partner-to-partner level, if we ask a partner to break the container, we enter into tricky territory. Sloppy container holding and breaches that are entirely consensual can have unintended long-term effects. For instance, both partners might be a “Yes” on the front end for the hit of instant gratification while remaining blissfully unaware that they’ve put a kink in their practice on the back end, the same way a building contractor might cut some corners to save on costs while introducing flaws. One or both partners may begin to get the feeling, no matter how subtle or quiet, that their partner is easily swayed and their confidence in the container grows weak by extension. Such a “loss of containment” tends to refuel the grip of the vigilance center. While many of us will experience pleasure and wonder at the beginning (and all stages) of our practice, as a human with a full range of emotions, sensations, etc. available, we will eventually feel more of everything. Sharing that experience with another person is one way to describe intimacy. A strong container we can trust enables us to feel safe enough to experience a fuller range of experiences in connection in and out of the nest. If a fuller, richer experience of life is one of the benefits you’d like from the practice, we suggest keeping a tight container. Further… Many people want to learn Orgasmic Meditation® asking for change, transformation, and more power in their lives and then want to change the external circumstances like the parameters of the practice. Our minds say, "If only this or that part of the practice was different, or this person or that thing would be more to my liking and comfort in this way or that, then I'd be happy and free!” But we know this isn't really how it works. Our willingness to be with and expand our ability to stay conscious in both pleasure and discomfort determines the quality of our lives. The whole idea of practice is that we have found something to surrender more and more deeply into regardless of whether the day brings pleasure or pain. Most of us have inherited and developed habits, thought forms, and preferences that limit our experience to a relatively narrow range or “comfort zone”. We refer to these unhelpful ways of thinking as conditioning or “crossed wires.” One way of thinking about the container of OM is as a set of consistently held external circumstances that we create so that we can enter, experience whatever the practice wants to show us, and have that internal landscape changed by it. In this way, we are the ones who get worked, find more personal freedom, power, and internal space for adjustment despite what may be happening at the point of connection in the nest or in our lives. We know deeply that freedom is available regardless of external circumstances. Keeping the container is our opportunity to practice and work those muscles and become stronger, more vulnerable, more sensitive, more willing to ask for what we want when it's deeply confronting to do so, more willing to be felt, etc., knowing that we’re inside something strong enough to hold us as we learn to be with the things we cannot control. Having a practice allows us to explore the great expanse of what’s possible with regularity. It is because of, not in spite of, the restrictions of the container that we can go increasingly deeper in orgasm. The notion of a container that fluctuates at the whim of negotiation between partners, while it might be fun, is antithetical to the benefits described above. The practice relies on our consistency and the knowledge that at no time will there be a request to change it or that OM will suddenly come off the table if such a request is denied. Imagine if we introduced a flexible, negotiable container and practitioners got reputations as "hard bargainers" or "easy pushovers." At that point, we'd be right back inside of our normal transactional conditioning all over again with nothing more than an excuse to play it all out under the guise of a practice. When we surrender in an OM, we’re not surrendering to our partner; we’re surrendering to the OM and to the orgasm state. Further yet… OneTaste® has no prescription for what people should do with their bodies or in their personal lives. We fully encourage consenting adults to do whatever they like, including containerless genital stroking if that’s what makes them happy. When we refer to Orgasmic Meditation®, we are making direct reference to the parameters of the container listed above that produce a specific set of benefits in both partners. Explicitly, simply because genitals are being stroked does not mean you are OMing. The container *is* what defines the activity as OM. Anything outside of those parameters is no longer OM and ought not to be referred to as such or conflated with OM.

Opting In and Opting Out

After you’ve said yes to an OM and have taken the steps to set a time, arrive, get into position, remove pants (for the strokee), etc., you can consider yourself “opted into” the experience of OM as described in this document free from modification. That means safeporting and stroking, as well as the elements of the practice that do not require safeporting (getting into position, putting on gloves, etc.) will occur as you and your partner move through the steps to OM outlined above. As with anything in life, either partner may always opt out of the experience at any time, and it is each person’s complete responsibility as an adult in an adult practice to state that overtly should they decide at any point that they do not want to proceed with an OM. As rare as it is that something should happen that would cause an OM to stop, it's crucial that both partners understand their role in both speaking and responding to each other should that occur. Should you find yourself in a situation where you need to opt out and stop the OM, you can use the same safe words you learned in your intro to OM or private training: red, yellow, and green. Red means that you need to stop and check in; yellow means that you’re feeling uncomfortable (which is actually oftentimes a good place to be!); and green means smooth sailing. If your partner happens to go to red during an OM, a good question to start with might be “what do you need?” Sometimes all a stroker might need to do is keep the finger very still without stroking for one or both partners to catch their breath. And, sometimes, we may need to stop the OM. In this case, talk through the experience and stay connected to discern the next best step.

Nest Etiquette

Post-OM processing of the experience is contrary to form, while extra physical contact (hugging, kissing, fondling, etc.) while in the nest is both contrary to form and container. The practice is already vulnerable enough for both people and quite the offer to say yes to. Adding additional weight to the experience reduces your likelihood of a repeat practice partner. Remember we all train each other! If I OM with Sally and Jenny, and Sally routinely wants to talk a bunch after OMs while Jenny is more of a “OM, be friendly and then leave - nothing extra“ kind of person, when I am wanting exactly what the practice offers in its "nothing extra" -ness, i.e., an experience of raw, unfiltered electricity and connection with another person, I am definitely calling Jenny. Sally has been relegated to my "call when you can say yes to a bunch of talking after the OM" list. People notice these things, and they do matter. Over time, pay attention to what habits your OM partners demonstrate that have you coming back for more versus the ones you're less likely to seek out again. Asking your partners for extra time to share experiences after the OM is complete, strokees pants are back on, and the nest is put away is always ok and, like the rest of life, may elicit a yes or no.

On Community

There are a number of grassroots communities throughout the world. Though OneTaste® publishes this document as well as training materials on the clitoral stroking practice it teaches, under the trademark    Orgasmic Meditation®, it does not manage or preside over these or any communities in any way. As with the varied and unique styles and flavors of yoga, meditation, and other mindfulness practices, it’s commonly understood that in the same way that an OM practice session or OM Circle is a physical practice space, the OM community is a practice-based community. As many people are new to practice, and even if they are not, they are new to OM, it’s suggested to take some time and learn the norms of any practice community, whichever you may explore.

Please Do Not…

There are a number of ways one could attempt to modify the practice and solicit for your own personal services or projects, engage community members in other practices by means of promotion, or engage in conversation about male genital stroking. Orgasmic Meditiation® has given rise to a consciousness-based clitoral stroking conversation and community. We all heard about a clitoral stroking practice and philosophy. We were educated about the benefits of clitoral stroking and how it benefits us all to stroke clitorises. We have perhaps seen a demonstration of clitoral stroking either online or in person. Based on what has drawn us all together, let there be no mistake about the purpose of these communities.

Orgasmic Meditation®, Orgasm, and Demonstrations of Orgasm

Orgasmic Meditation® is a clitoral stroking practice that cultivates the orgasm state. Demonstrations in all venues that feature a strokee on a demo table are not demonstrations of OM per se. Though they may be referred to as such from time to time and include many elements of OM, they are done by practitioners with various levels of experience in an art form located at the intersection of life and orgasm, known as the demonstration of orgasm. These practitioners have trained in a container referred to as Demo Training, which ranges in intensity from very light to quite rigorous. Thus, you may see techniques in demonstrations that do not belong in the OM practice. Note: OneTaste® does not certify OM Life® Certified Coaches to teach Demo Training forms (anything you don’t find in the standard OM container). Demo Training has been discontinued.

Additional Notes

- OM is a practice of “nothing extra”. Additions of music, eye-gazing, specific breathing techniques, being under the influence of alcohol or other substances while OMing, etc. aren’t part of the practice.

- When it comes to voice, strokees will find that with training and consistent practice over time, the sensation their voice produces will match the sensation in their genitals. It takes a while! The form for strokers is to direct their available attention and sensation they feel into their finger, not their voices. Attention should not be drawn to the experience the stroker is having.

- As OM is explicitly not sex and not a form of dating or romantic encounter, sexual accoutrement is not part of the practice. That means lingerie, or other provocative clothing, seductive behavior, or overtures such as roses, a bottle of wine, etc., no matter how positively intended, are not welcome and will likely taint the experience if attempted.

- Partners without clitorises are always strokers while partners with clitorises are almost always strokees. Partners with clitorises may receive training in the stroker role. Refer to OneTaste® online training or an OM Life® Certified Coach if you are interested in being trained in the stroker position.

- “Thank you” is a neutral response that acknowledges a communication was received. Noticing and adjustments can be capped with a “thank you,” indicating the communication was received.

- Regarding climax: in OM, we neither try to create it nor try to muffle it. If climax occurs during the course of the practice, that’s great! If it doesn’t, that's also great!

- Stroker always sits to the right of the strokee unless prevented by physical impairment (i.e., nerve damage, missing digits, etc.). In cases where strokers need alternate positions, they can refer to an OM Life® Certified Coach for guidance. Persons with disabilities that make it difficult to sit are also able to OM after learning alternate positions.

- The clitoris is the only part of the genitals being stroked. Stroking the labia, anus, or other parts of the anatomy, as well as using multiple fingers to stroke at any point during the OM, are considered container breaches. The exception is in the case where a strokee’s clitoris is too sensitive to touch. Most of the time we find that strokees have often been stroked too hard, and simply stroking softly remedies the situation. If not, the next step is to try either/both a) holding the finger still on the clitoris and experiencing the sensation at the point of connection or b) using a thick amount of lube on the forefinger and stroking the clitoris with the lube as opposed to the finger’s surface. As a last resort, stroking on the hood is permissible.

- Strokes occur in two directions: up and down; no other directions, i.e., circles or side to side.

- We’ve found that the 1 o’clock spot, located in the upper left-hand quadrant of the clitoris (strokee’s left), tends to be the location with the highest nerve concentration, and so we primarily focus our attention there.

- All adjustments are made verbally, i.e., no “let me show you” where the strokee directs the stroker physically with her hands.

- Standard etiquette in an OM is to limit talking to offers and requests: no sexy talk.

- Strokers and strokees want to relax their bodies as much as possible, i.e., no gyrating or moving hips.

- The stroker’s visual attention should remain on the genitals during the OM. Looking at the strokee’s face while safeporting and frames are the two routine exceptions.

- On the topic of sexual activity between OM partners, the form of the practice for sexually active couples is to finish the OM, strokee puts pants back on, nest is deconstructed, and supplies are cleaned up. The suggestion is to take a few minutes to fully punctuate the experience. And if you can, change context. Get a glass of water, leave the room, etc. The form is to avoid using OM as foreplay; your OM practice may begin to depend on whether you want to have sexual experiences afterwards. Beware of that which, no matter how innocuous it may seem, attaches itself to your practice!

- If you want to have two OMs back to back, complete the first OM including frames before beginning the second. Change gloves.

- Do your best to remove your OM practice from the context of your sex life, i.e., “We made out, then we OM’d, then we had sex” would be uncouth. In a home or hotel, OM on the floor, not on the bed, if possible.

- The gloves are a physical safety barrier and are a part of the practice. Don’t ask to use them.

- To prevent contamination of lube, do not use bare fingers in the lube jar. Always use a gloved finger in lube.

- No “double dipping.” Once a gloved finger has touched a woman’s genitals, it is not to be dipped back in the lube jar for more lube. Should this occur, the lube can be considered contaminated and should be disposed of.

Appendix A: Anatomy

• Anus

• Perineum: the area between the anus and the introitus

• Introitus: the entrance to the vagina, the area within the inner labia

• Labia majora: outer lips

• Labia minora: inner lips

• Clitoral hood: the labia minora meet at the top and form a hood covering the clitoris

• Clitoral glans (the "clit"): the highest concentration of nerve endings in the body. Its singular purpose is pleasure

• The 1 o’clock spot (or “the spot”): the upper-left quadrant of the glans, under the hood
